# Circular causality analysis of corporate performance and accounting quality in M&As

**DOI:** 10.1371/journal.pone.0308608

**Published:** 2024-10-17

**Authors:** Ionut Viorel Herghiligiu, Roxana Manuela Dicu, George-Marian Aevoae, Daniela Nicoleta Sahlian, Adriana Florina Popa, Ioan-Bogdan Robu

**Affiliations:** 1 Department of Engineering and Management, “Gheorghe Asachi” Technical University of Iași, Iași, Romania; 2 Academy of Romanian Scientists, 3 Ilfov, Bucharest, Romania; 3 Department of Accounting, Business Information Systems and Statistics, “Alexandru Ioan Cuza” University of Iasi, Iasi, Romania; 4 Department of Accounting and Audit, Bucharest University of Economic Studies, Bucharest, Romania; Yunnan Technology and Business University, CHINA

## Abstract

The past performance and the capital structure of the companies that are involved in mergers and acquisition (M&As) are considered into the analysis of the circular causality relationship between financial performance and market value. Considering two models, one for value relevance and one for accounting conservatism, this paper aims to analyze if the capital market influences the accounting practices of a target company or that the accounting figures influence the capital market. The analyzed sample used in the study is represented by the target companies involved in M&As which took place in the European Union Enlarged in 2017–2018. Financial and market data were considered for eight years (2011–2018). Using the conservatism model, the results show that targets’ earnings are significantly influenced by their financial leverage as an indicator for financial structure. Using the value relevance model, the capital market reaction is influenced by prices and return on equity that indicates the capital market influence on accounting figures.

## 1. Introduction

Companies use mergers and acquisitions (henceforth M&As) as external growth strategies, based on multiple motivations. Among the most obvious, the following could be mentioned: survival (suitable for companies with weak capitalization, in a defensive position or with a declining market share), diversification (applicable especially in the case of conglomerate operations or vertical mergers and acquisitions), protection (seeking a partner to counter situations such as the entry of a low-cost competitor, loss of patents or other innovations in favor of a competitor) or growth (involving companies in an offensive position, which intend to increase market share, sales or profitability).

Thus, M&As have always been associated with consolidating a company’s financial position and increasing its value, in order to improve the existing state or to correct some negative outcomes for the acquirers and the targets involved. As a result, firms involved in a concentration operation should benefit operationally and financially as a result of this activity [1,2], despite the number of failures in the field [3,4].

It could be noticed that literature focuses more on the outcomes of the M&As [5–9] and less on describing the companies, before the concentration, without correlating their performance with the post-M&A situation [10,11] or as a determinant for the decision to participate in concentration [12]. The purpose of this paper is to analyze, from an accounting point of view, European target companies’ past of financial and market activity, which led to their participation in external growth strategies.

In this regard, the research question of the paper is essential in describing the target companies which are of interest for the acquiring companies, because it describes a type of behavior: *what is first*? *Conservatism or value relevance*? in the case of the past performance of target companies involved in M&As at EU level.

The paper aim to analyze circular causality between financial performance and market value with the purpose to test what accounting feature is dominant, value relevance or conservatism. As consequence, for answering this research question, this paper starts with vector autoregression models (VAR).

In this study, the market-return relation for target companies involved in M&As is analyzed, for the years prior to the concentration, as determinants for the transactions. In this regard, we use price-to-book ratio (PBR) to describe the value that market participants attach to a company’s equity, relative to the book value of the company. Secondly, return on equity (ROE) was considered as being significant for the financial performance of the target companies, as a result of applying specific accounting practices, prior to M&A [13]. Considering the model proposed by Beaver *et al*. [14] and Basu’s [15] evidence regarding conditional conservatism or asymmetric timeliness, it was assessed if the accounting practices influence the market value of a company or if the capital market influences the accounting practices.

The sample used in the analysis is represented by the target companies which participated in 5.387 M&As that took place in the European Union enlarged (28 countries) in 2017–2018 period of time. The financial data for the selected companies covers eight years, from 2011–2018. The chosen period for the M&As is of interest because the years 2017–2018 were representative for Brexit. The European Union was tormented by Great Britain’s decision to leave and these two are comparable years, given that since March 29, 2019, the Great Britain wasn’t an EU member anymore. The chosen period for the financial and market data covers the period after the companies recovered from the financial crisis of 2008 (the post-crisis period) [16]. Secondly, starting from 2019–2020, the Covid-19 pandemic changed the dynamics of the companies, many of them stopping or partially working, depending on the core activity [17]. In terms of the data presented in the financial statements, it is subject to accounting principles, aimed at ensuring proper reporting to stakeholders. Starting from 2019 and continuing in 2020, the companies had to prepare the annual reports and the financial statements disclosing the Covid-19 impact, and also considering the going-concern concept [18–20].

In the study, autoregressive vectors (VARs) are first used to capture the relationship between return on equity (ROE) and price-to-book ratio (PBR), as they change over time. For each of the variables, a one year-lag of themselves was considered, to which we add return on assets (ROA) and financial leverage (FL). After determining the coefficients of the two VARs, Granger causality was used to determine which of the time series is forecasting the other one [21]. The results show that the market influences the performance of the target companies, prior to M&As (ROE is influenced by PBR).

For the next section of this research, structural equations models (SEM) were used to assess, estimate, and test a theoretical association of linear relations between the considered variables [22]. Using general linear models (GLM), a system of simultaneous equations was proposed. For the first one, the return on equity is a function of price-to-book ratio (the market-return model). Starting from the research of Basu [15], the conservatism model is considered to assess accounting earnings as a function of the unexpected returns, resulting from the market reactions to good or bad news. For the second one, the market reaction is a function of target company’s return (the return-market model or the value relevance model, given the fact that the accounting information is influencing the market and, implicitly, the decisions made by stakeholders) and it uses the estimate of the return from the first equation, given the results of the Granger testing. The result will show that, for the target companies, in the conservatism model, their decision to participate to M&As is influenced by the financial structure, while, in the value relevance model, the market reaction is significantly influenced by both their previous experience and the financial ratios (returns and financial leverage).

The paper is structured as follows. The *Literature review and hypotheses development* section is dedicated to the literature review on accounting quality in M&As, on value relevance and conservatism, which will lead to two distinct hypotheses. The *Data and methodology* paper section presents research methodology and design. The *Results and discussions* section presents the descriptive statistics and the results of the study, and the last section concludes.

## 2.Literature review and hypotheses development

There are different theories which describe the motives for M&As, domestic or cross-border, most of them described by Buckley *et al*. [23], Trautwein [24], Aevoae and Georgescu [25], Maha *et al*. [26] and Kapil and Dhingra [27]. According to Buckley *et al* [23], cross-border M&As are the main part of the foreign direct investment (henceforth FDI), the latter, as a total, being determined by four purposes including search for new market, search for an asset, achieving efficiency, and exploiting natural resource. On the same lines, Trautwein [24] stated that M&As are driven by empire building model, efficiency model, valuation model, process model and monopoly model. Dunning [28] proposes a theory according to which an acquirer penetrates foreign market seeking solely the location advantages (the eclectic theory). There are researchers who disagree with Dunning by stating that his theory is insufficient to explain M&A occurrence. Mathews [29] proposed the LLL (linkage, learning & leverage) model in this regard, considering that strategic assets are the main influence factor for an M&A. The Uppsala theory, proposed by Johanson and Vahlne [30], although related to networks of relationships between firms, can explain the tendency of domestic firms to test international markets.

Given the diversity and popularity of M&As as external growth strategies, the literature on the matter focused, over decades, on various issues related to the involved companies and their performance [31–35], the vast majority of them focusing on the performance on the financial market, under the form of abnormal returns (up to 41% of the studies). On the other hand, Thanos and Papadakis [36] bring into attention the accounting-based measures as a viable metric of M&A performance, while Zollo and Meier [31] bring into attention the subjective performance, related to synergy realization and reaching strategic objectives. Thus, both the market and the accounting figures may be of great importance when assessing the transaction itself but also the companies involved.

Performance as an influence factor for M&As is a subject of interest in literature and it is analyzed both as a determinant and as a consequence of the transaction. The perspective on performance can vary, depending on the authors’ perspective regarding the timeline of the involved companies and the used measures. Considering the post-M&A period, Grigorieva and Petrunina [37] focused their perspective on two types of studies–event studies and accounting studies. Papadakis and Thanos [38] added to their analysis the managers’ subjective assessments, as a perspective on the performance of the concentration.

Although the results are inconsistent (for example, returns to acquiring firms are sometimes positive, sometimes negative, and sometimes equal to zero), the event study methodology is a commonly used one for assessing the performance of the companies involved in M&As [39,40]. Regarding the accounting-based measures–sales, book value of equity, market value, cash-flow and others–these offer a positive perspective on M&As performance [41,42], while the use of ratios and return lead to a decline in margins and thus to a poor perspective on post-M&A performance [43,44]. Thanos and Papadakis [36] present three major categories of accounting measures, found in literature: *ratios* (return on sales, return on investment, return on equity and return on capital employed), *growth measures* (sales and profits, assets–usually using logarithmic scale) and *operating cash-flows*, as an image of a company’s liquidity.

Another perspective on performance considers the values reported by the participating companies before the transaction (pre-M&A), taking into account the same accounting measures as accounting determinants or influence factors for the M&A [36]. Why do accounting figures play such an important role in the decision-making process regarding these growth operations?

Bartov et al. [45] analyze the overbidding phenomenon in M&As and the role of well-documented accounting figures in modelling optimal bidding for the acquirer. According to Rau and Vermaelen [46] and Ismail et al. [47], acquiring companies with poor track record or with high book-to-market ratio tend to be more prudent and are not motivated by hubris when approving an acquisition, while a bidder with low book-to-market ratios ("glamour firm") overestimates its abilities and hence approve the acquisition. There are authors [10,48] which correlate pre- and post-performance of companies involved in M&As, considering research productivity, return on investment, and profit margin.

Marquardt and Zur [49] discuss the influence of target’s accounting quality on the parameters of the transaction and their results show positive impact on its speed and the completion of the deal. But which are the drivers and the characteristics of the accounting quality?

According to Dechow *et al*. [50], the quality of the firm’s earnings depends on both the firm’s fundamental performance and on the accounting system that measures it. Thus, the issue related to accounting quality in M&As can be also analyzed considering the accounting standards used by both acquiring and acquired companies, when considering investing in foreign markets and, implicitly, participating in cross-border M&As. Considering the premise that the use of International Financial Reporting Standard (IFRS) leads to a higher information quality [51–53], Francis *et al*. [54] asserted that the mandatory adoption of the international referential in 2005 led to an increase in M&A activity between countries which use this reporting system.

There are two antagonist concepts, proposed by IASB’s Conceptual Framework for Financial Reporting, related to financial information quality: value relevance and conservatism or prudence [55]. Although the last two concepts are usually interchangeable, Barker [56] makes a distinction between them, considering that ‘conservatism’ refers to any method of accounting that leads to book value being less than economic value, while ‘prudence’ is a specific type of conservatism arising from a ‘cautious’ response to uncertainty. In M&A literature, the “prudence effect” also means that when uncertainty increases, the sensitivity of enterprises rises, and small changes in some factors in the market can also be sensed, such as supply and demand requirements and commodity prices [57,58]. Simo *et al*. [59] note that conservatism should be viewed more as a state of mind, translated into a careful assessment of all possible uncertainties and risks, than a systematic calculation. By associating it with risk management, the pessimism resulting from the use of conservatism tempers the optimism of managers, its purpose being to maintain shareholder confidence [60]. As a result, studies show that earnings conservatism is more pronounced in common-law developed countries and in those applying IFRS [61].

Basu [15] interprets conservatism by considering the faster reaction of earnings to bad news, compared to good news, both reflected in the stock returns, approach that will be used in this paper. We describe the reaction of accounting data provided by the company, in terms of return on equity (ROE), in sufficiently adverse circumstances dictated by the market. This is known as conditional conservatism, also discussed by Ball *et al*. [62], Ryan [63] and Lara *et al*. [64], compared to unconditional one [14], which is reflected in lower book values of equity and assets. Also, Basu’s paper can be considered a value relevance study because it examines the relationship between earnings (which is a product of applying specific accounting practices) and market value (reflected in the returns). Although Basu’s study has had a substantial impact on the accounting research, there are also studies like the ones of Patatoukas and Thomas [65], Dietrich *et al*. [66] and Givoly *et al*. [67], which provide considerable evidence suggesting that the differential timeliness measure is associated with a variety of substantial biases. Despite these results, we will assume that the market affects the accounting performance and earnings, in order to describe our sample analysis.

As it could be noticed, the market is very important for analyzing the influence of information on the decision-making process of the stakeholders. The same author, but also Khan and Watts [88], suggested a significant reduction in accounting conservatism, after IFRS adoption, with opposite effects on the performance reported by the companies and on the value relevance, because the timely recognition of all the events could lead to a more reliable information [68].

Brown Jr *et al*. [69] define value relevance as the summary provided by accounting earnings about information possessed in market prices, while Karğin [70] provide a more accurate definition. In his opinion, value relevance is defined as the ability of information disclosed by financial statements to capture and summarize firm value on the market. In this regard, it can be used as an association between market values and accounting values [71], an ability of accounting information to capture or summarize information affecting share price or stock return [72]. In completion to this opinion, Barth *et al*. [73,74] assert that value relevance research is a basis for establishing that some financial accounting practices are perceived by equity investors as conservative, like the amounts relating to intangible assets, growth opportunities and alternative performance measures.

The performance, both from an accounting and market perspective, was the subject of analysis over the years, from different approaches and without a consensus on the results. Without analysing a possible causality between accounting and market performance measures, Merchant [75] assesses the management performance though these indicators, while Hamdan [76] argues that the financial performance positively impacts on intellectual capital, whilst financial market has the opposite effect. Hax [77] argues that accounting performance is directly correlated to market performance, and a point drop in market performance can lead to hostile takeovers. The relationship between accounting and market measures of firm performance was also a subject of interest for Gentry and Shen [78], who found them to be positively correlated. The contribution of our study is that it expands the analysis of causality between financial and market performance to the target companies involved in M&As, as a better way to describe a possible pattern for these companies.

Given the importance and the influence of both conditional conservatism and value relevance in the decision-making process, in the case related to participating to external growth strategies like M&As, the following general hypothesis and the subsequent specific hypotheses are proposed to be tested and validated:

H_G_: At M&A level, there is a circular causality relationship between financial performance (return on equity), and market price (price-to-book ratio), as endogenous variables, considering the influence of two exogenous variables (return on assets, and financial leverage).

Essentially, this general hypothesis can be separated into two different working hypotheses, and the proposed analysis will establish which of the ratios first influences the other one, considering the accounting values and the market values. The analysis will also establish if the influence is significant or not,

Hs_1_: The performance of a target company, prior to M&A, is significantly influenced by the market prices.

Hs_2_: The target companies’ value on the market is significantly influenced by the performance reflected in the accounting information.

The main objective of our study is to identify which of the two endogenous variables (return on equity, and price-to-book ratio) represent the *causa causarum* in the circular causality relationship proposed in the study, as well as the influence of the determinant factors on them. Thus, the study adds up to past literature concerning the causality between financial and market performance.

The hypotheses will be tested and validated using statistical software SPSS 25.0 and Stata 15.1.

## 3. Data and methodology

### 3.1. Target population and analyzed sample

To test and to validate the two proposed research hypotheses, the study analyses the empirical data related to 5387 M&As, for the 2017–2018 period of time, considering the target companies located in the European Union enlarged (28 countries). The choice for the period was based on the fact that, according to Institute of Mergers, acquisitions and Alliances [79], the largest number of transactions was recorded in the proposed years (52.740 M&As in 2017 and 50.607 M&As in 2018). The data source was Zephyr database. The financial data used in analysis was considered for eight years, from 2011–2018, and it was collected from Orbis database.

The years 2017–2018 were significant for the European Union. On January 26, 2017, in British Parliament, the European Union Bill (Notification of Withdrawal) was introduced, a 137-word bill which gave Theresa May the power to start the Brexit process by triggering Article 50 of the Treaty on European Union. The year 2018 marked a historical moment for the European Union, because on 14 of November, the Brexit Withdrawal Agreement was published, endorsed on 25 of November by 27 EU member states. The act, covering matters as money, citizens’ rights, border arrangements and dispute resolutions, had great impact on the economy of the EU, including Romania [80,81]. As a result of Brexit, financial services and financial technology moved from London to other financial centres in EU [82], which led to a destabilisation of financial markets [83]. Thus, a decline in the number and value of M&As was observed in 2019, compared to previous years [84,85]. Also, the start of Covid-19 pandemic in 2019 makes it an atypical year, which necessitates a special analysis.

### 3.2. Description of variables and the proposed models

Starting from Beaver et al. [86] that use OLS and 2SLS in order to study the “effect of simultaneity in a piecewise linear system of earnings and return” in our research we proposed to analyze the relationship between return on equity (ROE) and price-to-book ratio (PBR), as they change over time, using vector autoregression (VAR). The proposed equations are as follows:

$${PBR}_{t}=\beta_{0}+\sum_{i=1}^{t}\beta_{1}∙{PBR}_{t-i}+\sum_{i=0}^{t}\beta_{2}∙{ROE}_{t-i}+\sum_{i=0}^{t}\beta_{3}∙{ROA}_{t-i}+\sum_{i=0}^{t}\beta_{4}∙{FL}_{t-i}+\varepsilon_{i}$$ (1)

$${ROE}_{t}=\gamma_{0}+\sum_{i=0}^{t}\gamma_{1}∙{PBR}_{t-i}+\sum_{i=1}^{t}\gamma_{2}∙{ROE}_{t-i}+\sum_{i=0}^{t}\gamma_{3}∙{ROA}_{t-i}+\sum_{i=0}^{t}\gamma_{4}∙{FL}_{t-i}+\varepsilon_{i}$$ (2)

where:

*PBR*_*t*_ is the ratio between the market capitalisation of the target company and the book value of the same company, for year *t*;

*PBR*_*t*−*i*_ is the ratio between the market capitalization of the target company and the book value of the same company, considering a one-year lag *t-i*;

*ROE*_*t*_ represents the return on equity between the net income and the total shareholders’ equity for the target companies, for year *t*;

*ROE*_*t*−*i*_ represents the return on equity for the current year *t*, and the previous ones as ratio between the net income and the total shareholders’ equity for the target companies;

*ROA*_*t*−*i*_ shows how profitable (net income) a target company is relative to its total assets, for the *t-i* years;

*FL*_*t*−*i*_ is the financial leverage, calculated as the ratio between the total liabilities of the target company and the shareholders’ equity, and includes long-term bearing debts, considering a one-year lag *t-i*.

After applying the models to our sample of data, we apply Granger test [21]. Its causality is often used as a prediction statistical concept, where, if *PBR*_*t*_ were to “granger-causes” a signal to *ROE*_*t*_, then the past information of *PBR*_*t*_ should help in predicting *ROE*_*t*_ more than the values contained in the past values of *ROE*_*t*_ alone.

$${ROE}_{t}=f({PBR}_{t-1};\;{ROE}_{t-1};\;{ROA}_{t};\;{FL}_{t})+\varepsilon_{t}$$ (3)

Using GLM, we propose the *market-return model (conservatism model)*, as follows:

$${ROE}_{t}=\delta_{0}{+\delta_{1}∙PBR}_{t-1}+\delta_{2}∙{ROE}_{t-1}+\delta_{3}∙{ROA}_{t}+\delta_{4}∙{FL}_{t}+Time\;fixed\;effects+\varepsilon_{t}$$ (4)

We use the following as a function for value relevance of financial information:

$${PBR}_{t}=f({PBR}_{t-1};\;{ROEest}_{t-1};\;{ROA}_{t};\;{FL}_{t})+\varepsilon$$ (5)

The return-market model (value relevance model) is:

$${PBR}_{t}=\theta_{0}{+\theta_{1}∙PBR}_{t-1}+\theta_{2}∙{ROEest}_{t-1}+\theta_{3}∙{ROA}_{t}+\theta_{4}∙{FL}_{t}+Time\;fixed\;effects+\varepsilon_{t}$$ (6)

where:

*ROEest*_*t*−1_–represents the estimated value for the return on equity for year *t*, based on conservatism model.

The results will be presented in the next section, considering the financial data for 2011–2018 period of time.

## 4. Results and discussions

The descriptive statistics for the selected data sample are presented in Appendix 1 in S1 File. The variables that were analyzed are *ROE*, *ROA*, *FL* and *PBR* for which there were estimated the mean, standard deviation, confidence interval, minimum and maximum values–period 2011–2018.

Considering the analyzed period (before Covid-19 pandemic crisis), using ANOVA method–polynomial model, in this study there were estimated the significant differences between the values of analyzed variables for 2012–2018 period (see Appendix 2 in S1 File). From Appendix 2 in S1 File it can be observed that there are significant differences in evolution of the analyzed variables for the period 2012–2018.

Using Post Hoc Tests (LSD) for multiple comparisons, the significant differences by period for each variable were estimated and the results are presented in Appendix 3–6 in S1 File. From tables presented in the appendices mentioned before it can be observed that 2017 and 2018 financial years had a significant influence on the analyzed variables evolution.

The correlations between analyzed variables are presented in Appendix 7 in S1 File. From the table included in this appendix it can be observed that there are positive and significant correlations between *ROE* and *FL*, *ROE* and *PBR*, *ROA* and *FL*, *ROA* and *PBR*, *FL* and *PBR*.

To respond to the research question of the paper, which refers to: *what is first*? *Conservatism or relevance*? in the case of past performance of target companies involved in M&As at EU level, this paper starts with vector autoregression models (VAR). The estimated coefficients are presented in Table 1. Likewise, the data from Table 1 provide the test results associated to the determined lagged, for the variables includes in the models.

**Table 1:** The coefficients for VARs.

	**PBR**			
	**Coef**	**Std.Err.**	**Z**	**P>Z**
PBR L.1	-0.0497	0.13429	-0.37	0.711
ROE L.1	0.35053	1.07026	0.33	0.743
ROA	19.01*	7.36716	2.58	0.010
FL	0.75958**	0.185	4.11	0.000
Intercept	-6.1721*	2.41166	-2.56	0.010
	**ROE**			
	**Coef**	**Std.Err.**	**Z**	**P>Z**
PBR L.1	2.49731**	0.17952	13.91	0.000
ROE L.1	5.91759**	1.43069	4.14	0.000
ROA	21.1902*	9.84815	2.15	0.031
FL	1.13717**	0.2473	4.6	0.000
Intercept	-14.977**	3.22382	-4.65	0.000

** and * indicate the statistical significance at 1 and 5% level, respectively.

With an R-square of 0.8901, the model where price-to-book ratio is dependent variable (the value relevance model) is significant and the independent variables explain 89.01% of the variance of the dependent variable. In the case of the second model (the conservatism model), the one where the return on equity is considered dependent variable, the R-square is 0.9773, which means that the chosen independent variable explain a great percentage of the variance of the variable considering a significance level of 1%.

The Granger test of causality, presented in Table 2, shows that return on equity (ROE) is first influenced by PBR, which can be translated in the fact that, at the analyzed sample’s level, the market influences the performance of the target companies, and implicitly, their accounting practices. The market influences could be determined by financial, pandemic, energies, social and political crises with direct impact on investor’s decisions on financial markets.

**Table 2:** The Granger causality test.

Equation	Excluded	chi2	df Prob	> chi2
PBR	ROE	0.10727	1	0.743
PBR	ALL	0.10727	1	0.743
ROE	PBR	193.52*	1	0.000
ROE	ALL	193.52*	1	0.000

* Indicate the statistical significance at 1% level.

The results of the structural equations modelling are presented in Table 3 (for conservatism model) and Table 4 (for value relevance model).

**Table 3:** The parameter estimates for the conservatism model.

Parameter	B	Std. Error	t	95% Confidence Interval	
				Lower Bound	Upper Bound
Intercept	8.530	2.217	3.848* (0.000)	4.185	12.875
ROEt-1	0.018	0.146	0.121 (0.904)	-0.268	0.303
PBRt-1	0.002	0.040	0.040 (0.968)	-0.078	0.081
ROAt	-1.707	1.491	-1.144 (0.252)	-4.630	1.217
FLt	0.157	0.048	3.238 (0.001)	0.062	0.251
[Time = 2012]	-8.364	3.014	-2.775* (0.006)	-14.271	-2.456
[Time = 2013]	-8.359	3.014	-2.773* (0.006)	-14.267	-2.451
[Time = 2014]	-8.152	3.013	-2.705* (0.007)	-14.059	-2.246
[Time = 2015]	-8.155	3.014	-2.706* (0.007)	-14.062	-2.248
[Time = 2016]	-8.237	3.013	-2.733* (0.006)	-14.143	-2.330
[Time = 2017]	-8.361	3.015	-2.773* (0.006)	-14.271	-2.451
[Time = 2018]	0a	.	.	.	.

**Table 4:** The parameter estimates for the value relevance model.

Parameter	B	Std. Error	t	95% Confidence Interval	
				Lower Bound	Upper Bound
Intercept	0.698	0.334	2.087** (0.037)	0.042	1.353
PBRt-1	0.367	0.006	59.738*** (0.000)	0.355	0.379
ROEestt-1	-1.142	0.054	-20.966*** (0.000)	-1.249	-1.035
ROAt	-0.415	0.227	-1.830* (0.067)	-0.859	0.029
FLt	0.454	0.007	62.300*** (0.000)	0.440	0.469
[Time = 2012]	-0.054	0.455	-0.120 (0.905)	-0.946	0.837
[Time = 2013]	0.160	0.455	0.352 (0.725)	-0.731	1.051
[Time = 2014]	-0.620	0.455	-1.363 (0.173)	-1.510	0.271
[Time = 2015]	0.701	0.455	1.542 (0.123)	-0.190	1.592
[Time = 2016]	-1.042	0.454	-2.293** (0.022)	-1.933	-0.152
[Time = 2017]	1.411	0.455	3.102*** (0.002)	0.519	2.302
[Time = 2018]	0a	.	.	.	.

***, ** and * indicate the statistical significance at 1%, 5% and 10% level, respectively.

From Table 3, considering the Basu’s [15] interpretation of conservatism, based on earnings-on-market’s returns, as a result of bad news/ good news (financial, pandemic, energies, social and political crises with direct impact on investor’s decisions on financial markets) it could be observed that, in the case of the analyzed sample, the variables included in the model do not influence each other. However, for target companies, the capital structure is significant for the investor’s interest, being measured by financial leverage ratio. This means that the main trigger for participating in M&As for target companies is their financial structure, more precisely the value of their debts compared with the company total equities. Although neoclassical theory suggests that a firm’s investment decisions are unaffected by its financial conditions because external and internal funds are perfect substitutes within perfect capital markets, information asymmetry and the conflicts of interest between corporate insiders and outside investors render such capital market perfection unlikely. Thus, the financing constraints play an important part in the decision to participate in M&As, which makes the results consistent with those of Chen et al. [87] and Khatami et al. [88]. Also, from Table 3 there are significant time fixed effects on the conservatism model.

The value relevance model explains that accounting information is relevant for investors and could influence their decisions on the capital market. Based on this assumption, the market reacts to accounting information that is reported by the target companies as a result of applying specific accounting practices and rules. When applying the value relevance model, the results show that the PBR for the current year is influenced not only by the values from previous years (t = 59.738, sig. = 0.000), but also by the financial returns (ROE and ROA) which result from the financial statements that are reported by the target companies and also by the capital structure (which is assessed using FL). The choice for PBR is consistent with the one of Khan and Watts [89]. Using the returns for assessing the performance of the M&As is consistent with the studies of Bionda et al. [11] and Grigorieva and Petrunina [37].

For the value relevance model these results show that investor’s decisions on capital market are significantly influenced by companies’ performance (assessed by ROA and ROE) and the degree of indebtedness with direct impact on going concerned assumption. The importance of returns in M&A process is also stated by Ismail et al. [47] and Liu et al. [10].

As studies show, there are two types of analyses researchers employ when assessing the performance of M&As: event studies (based on abnormal returns) and accounting performance studies (based on financial performance reported by the companies in their annual reports). In our study, we intend to create a “performance timeline” for the target companies involved in this type of operations, by establishing the causality between market performance (reflected in PBR) and accounting performance (reflected in ROE). As the results show, the accounting performance is firstly influenced by the market, but there is no significant influence of the variables, except for the financial leverage, which leads us to conclude that the ratio between debts and equity is of highly importance for the companies involved. In the second model, we notice a significant influence of the accounting figures on the image of the company on the market, which leads to the conclusion that the market reacts to the financial reports of the target companies.

## 5. Conclusions

In mergers and acquisitions, investors are interested in the financial performance and position of target companies, but also, they take into consideration the news that came from the capital market. In this context, given the number of studies which focus on accounting and market factors which may be of interest for the acquirers in M&As, this study assessed and estimated which of the two performance measures influenced the other (accounting figures or news from capital market) using structural equations models that include a conservatism model and a value relevance model, similar to the ones of Beaver et al. [86,90].

Both conservatism and value relevance are characteristics of financial information which may affect the decision-making process. Financial accounting is inherently conservative, which leads to a net value of assets and a book value being less than their economic value (unconditional conservatism). Also, the book value of equity may be reported due to adverse circumstances, which may occur from the specific environment and the market characteristics. The decision of the International Accounting Standards Boards (IASB) to introduce conservatism in the Conceptual Framework of International Financial Reporting Standards (IFRS) in 2018 was a signal for the companies to exercise more caution when making accounting judgments and estimates under uncertainty conditions. At the same time, the value relevance of accounting information from financial statements reflects the information disclosed capacity by the companies to capture and summaries firm value, its financial performance and capital structure.

Considering the return on equity as representative for companies’ performance and price-to-book ratio a consequence of the market reaction to the accounting figures presented by the companies, this study used Beaver et al. [14] and Basu’s [15] models to analyze the circular causalities between financial return and market reaction, using VAR and SEM.

Applying VAR, it was identified that market influenced the financial performance of the target companies involved in M&As in the EU enlarged (28 countries), during 2017–2018 interval. The Granger causality test allowed to investigate the circularities between analyzed variables and to propose the structural equation models (SEM) (see Eqs 4 and 5)–which included two separate models.

The first model, associated to conditional accounting conservatism, assessed the influence of the market’s PBR on ROE, creating a market-to-return model and considering that the financial markets send signals into accounting practice, with direct impact on accounting figures and implicitly on financial performance [15]. Also, the first model showed that in the analyzed sample of target companies, the financial structure was a significant factor in the decision-making process of investors to participate in external growth strategies, like M&As.

For the second model, the dependent variable from the conservatism model (ROE) became independent variable and PBR became dependent variable, using the value relevance model. According to this model, the accounting information reported by the companies together with the market prices from the previous year significantly influences current price-to-book ratios.

These results indicated that, according to conservatism model, the market influences the financial performance of the target companies, but the value relevance associated to accounting figures is the one that could change the market perception regarding the information that are reported by the companies.

The study has practical implications for practitioners and policy makers, because, for a consistent sample of M&As (5387 deals), it states that the financial market significantly reacts to the accounting figures publicly presented by the target companies involved in transactions, namely the returns and the financial leverage, for the years prior the deal (for the value relevance model), although the results show that the market influences the performance of the target companies, prior the M&As (ROE is influences by the PBR). The market influences could be determined by financial, pandemic, energies, social and political crises with direct impact on investor’s decisions on financial markets.

One of the limits of the study arises from not analyzing the influence of the results on some key characteristics of the M&As in the sample of analysis. Also, limited or poor disclosure of companies, especially small and medium ones, may influence the volume of information regarding financial markets.

Finally, several questions arise. Future research could further examine if the results are consistent when using other financial markets indicators (Tobin’s Q, dividend yield or market capitalization). Furthermore, considering the same sample of companies, two questions raised are whether the financial market indicators or the capital structure of the target companies affect the acquirers’ equity ownership choices and amounts to be invested.

## Supporting information

S1 File (DOCX)
