# Supplementary material for: Circular causality analysis of corporate performance and accounting quality in M&As
Source: PLoS One. 2024 Oct 17;19(10):e0308608. doi: 10.1371/journal.pone.0308608 (PMC11486431; doi:10.1371/journal.pone.0308608)
Supplement: S1 File — (DOCX) [file pone.0308608.s001.docx]

# Annexes

**Appendix 1.** Descriptive statistics

|  | | **N** | **Mean** | **Std. Deviation** | **Std. Error** | **95% Confidence Interval for Mean** | | **Minimum** | **Maximum** |
| --- | --- | --- | --- | --- | --- | --- | --- | --- | --- |
|  |  |  |  |  |  | **Lower Bound** | **Upper Bound** |  |  |
| ROE | 2012 | 5387 | -0.0876 | 1.3587 | 0.0185 | -0.1239 | -0.0513 | -20.8699 | 32.8482 |
|  | 2013 | 5387 | -0.0354 | 13.2087 | 0.1799 | -0.3882 | 0.3173 | -513.0000 | 184.6923 |
|  | 2014 | 5387 | 0.1213 | 7.1843 | 0.0978 | -0.0705 | 0.3132 | -50.8922 | 510.5238 |
|  | 2015 | 5387 | -0.0491 | 1.1220 | 0.0152 | -0.0790 | -0.0191 | -23.9693 | 16.4936 |
|  | 2016 | 5387 | -0.0846 | 1.1148 | 0.0151 | -0.1144 | -0.0549 | -16.3429 | 16.1082 |
|  | 2017 | 5387 | 0.1374 | 1.5730 | 0.0214 | 0.0954 | 0.1794 | -65.7143 | 30.5536 |
|  | 2018 | 5387 | 8.3250 | 413.2282 | 5.6301053 | -2.7122 | 19.3623 | -449.1725 | 30055.0000 |
|  | Total | 37709 | 1.1895 | 156.3068 | 0.8049 | -0.3881 | 2.7672 | -513.0000 | 30055.0000 |
| ROA | 2012 | 5387 | 0.3435 | 0.2964 | 0.0040 | 0.3356 | 0.3514 | -1.0464 | 3.3219 |
|  | 2013 | 5387 | 0.3512 | 0.3169 | 0.0043 | 0.3428 | 0.3597 | -0.1029 | 4.0316 |
|  | 2014 | 5387 | 0.3376 | 0.2724 | 0.0037 | 0.3303 | 0.3449 | -0.5482 | 2.1671 |
|  | 2015 | 5387 | 0.3766 | 1.1157 | 0.0152 | 0.3468 | 0.4064 | -0.4451 | 77.7995 |
|  | 2016 | 5387 | 0.3632 | .03690 | 0.0050 | 0.3533 | 0.3730 | -1.7124 | 2.3649 |
|  | 2017 | 5387 | 0.3969 | 0.4958 | 0.0067 | 0.3836 | 0.4101 | -0.1288 | 4.0948 |
|  | 2018 | 5387 | 0.3889 | 0.3891 | 0.0053 | 0.3785 | 0.3993 | -0.3232 | 2.6098 |
|  | Total | 37709 | 0.3654 | 0.5403 | 0.0027 | 0.3600 | 0.3709 | -1.7124 | 77.7995 |
| FL | 2012 | 5387 | 2.0768 | 7.6546 | 0.1042 | 1.8723 | 2.2812 | -131.2088 | 264.2174 |
|  | 2013 | 5387 | 2.4990 | 10.4260 | 0.1420 | 2.2205 | 2.7775 | -260.9331 | 210.6154 |
|  | 2014 | 5387 | 2.0199 | 17.9755 | 0.2449 | 1.5397 | 2.5000 | -724.1317 | 340.4878 |
|  | 2015 | 5387 | 1.3614 | 18.9703 | 0.2584 | 0.8547 | 1.8681 | -422.6737 | 175.5270 |
|  | 2016 | 5387 | 1.5229 | 10.5455 | 0.1436 | 1.2412 | 1.8046 | -204.2857 | 63.6400 |
|  | 2017 | 5387 | 4.1176 | 9.0506 | 0.1233 | 3.8758 | 4.3593 | -23.9373 | 172.6364 |
|  | 2018 | 5387 | 2.8681 | 29.9581 | 0.4081 | 2.0679 | 3.6683 | -1521.4545 | 129.6875 |
|  | Total | 37709 | 2.3522 | 16.6727 | 0.0858 | 2.1839 | 2.5205 | -1521.4545 | 340.4878 |
| PBR | 2012 | 5387 | 1.7076 | 6.0697 | 0.0826 | 1.5455 | 1.8697 | -83.6169 | 197.6162 |
|  | 2013 | 5387 | 2.5748 | 14.5272 | 0.1979 | 2.1868 | 2.9628 | -50.7624 | 481.5089 |
|  | 2014 | 5387 | 1.8417 | 43.8975 | 0.5980 | 0.6692 | 3.0142 | -2429.9960 | 895.2319 |
|  | 2015 | 5387 | 2.3987 | 16.8686 | 0.2298 | 1.9482 | 2.8493 | -75.8078 | 593.0482 |
|  | 2016 | 5387 | 1.1335 | 20.9154 | 0.2849 | 0.5749 | 1.6922 | -351.1513 | 452.4148 |
|  | 2017 | 5387 | 4.3277 | 14.8466 | 0.2022 | 3.9311 | 4.7242 | -182.7706 | 593.4947 |
|  | 2018 | 5387 | 3.2716 | 39.3801 | 0.5365 | 2.2198 | 4.3235 | -189.5170 | 2805.4300 |
|  | Total | 37709 | 2.4651 | 25.8408 | 0.1330 | 2.2043 | 2.7259 | -2429.9960 | 2805.4300 |

**Appendix 2.** ANOVA results (polynomial for the analyzed period)

|  | | | | **Sum of Squares** | **df** | **Mean Square** | **F** | **Sig.** |
| --- | --- | --- | --- | --- | --- | --- | --- | --- |
| ROE | Between Groups | (Combined) | | 320272.332 | 6 | 53378.722 | 2.185 | 0.041 |
|  |  | Linear Term | Contrast | 123909.400 | 1 | 123909.400 | 5.073 | 0.024 |
|  |  |  | Deviation | 196362.932 | 5 | 39272.586 | 1,608 | 0.154 |
|  | Within Groups | | | 920954800.321 | 37702 | 24427.213 |  |  |
|  | Total | | | 921275072.654 | 37708 |  |  |  |
| ROA t | Between Groups | (Combined) | | 16.835 | 6 | 2.806 | 9.624 | 0.000 |
|  |  | Linear Term | Contrast | 12.313 | 1 | 12.313 | 42,232 | 0.000 |
|  |  |  | Deviation | 4.523 | 5 | .905 | 3.102 | 0.008 |
|  | Within Groups | | | 10992.139 | 37702 | .292 |  |  |
|  | Total | | | 11008.974 | 37708 |  |  |  |
| FL | Between Groups | (Combined) | | 28335.548 | 6 | 4722.591 | 17.032 | 0.000 |
|  |  | Linear Term | Contrast | 5031.931 | 1 | 5031.931 | 18.148 | 0.000 |
|  |  |  | Deviation | 23303.617 | 5 | 4660.723 | 16.809 | 0.000 |
|  | Within Groups | | | 10453706.892 | 37702 | 277.272 |  |  |
|  | Total | | | 10482042.440 | 37708 |  |  |  |
| PBR | Between Groups | (Combined) | | 37017.519 | 6 | 6169.586 | 9.252 | 0.000 |
|  |  | Linear Term | Contrast | 10792.388 | 1 | 10792.388 | 16.184 | 0.000 |
|  |  |  | Deviation | 26225.130 | 5 | 5245.026 | 7.865 | 0.000 |
|  | Within Groups | | | 25142447.882 | 37702 | 666.873 |  |  |
|  | Total | | | 25179465.401 | 37708 |  |  |  |

**Appendix 3.** Post Hoc Tests (LSD) for multiple Comparisons: ROE

| **(I) Time** | **(J) Time** | **Mean Difference**  **(I-J)** | **Std. Error** | **Sig.** | **95% Confidence Interval** | |
| --- | --- | --- | --- | --- | --- | --- |
|  |  |  |  |  | **Lower Bound** | **Upper Bound** |
| 2012 | 2013 | -0.0522 | 3.0114695 | 0.986 | -5.9547 | 5.8503 |
|  | 2014 | -0.2090 | 3.0114695 | 0.945 | -6.1115 | 5.6935 |
|  | 2015 | -0.0385 | 3.0114695 | 0.990 | -5.9411 | 5.8639 |
|  | 2016 | -0.0030 | 3.0114695 | 0.999 | -5.9055 | 5.8995 |
|  | 2017 | -0.2251 | 3.0114695 | 0.940 | -6.1277 | 5.6773 |
|  | 2018 | -8.4127* | 3.0114695 | 0.005 | -14.3152 | -2.5101 |
| 2013 | 2012 | 0.0522 | 3.0114695 | 0.986 | -5.8503 | 5.9547 |
|  | 2014 | -0.1568 | 3.0114695 | 0.958 | -6.0593 | 5.7457 |
|  | 2015 | 0.0136 | 3.0114695 | 0.996 | -5.8889 | 5.9161 |
|  | 2016 | 0.0492 | 3.0114695 | 0.987 | -5.8533 | 5.9517 |
|  | 2017 | -0.1729 | 3.0114695 | 0.954 | -6.0755 | 5.7296 |
|  | 2018 | -8.3604* | 3.0114695 | 0.006 | -14.2630 | -2.4579 |
| 2014 | 2012 | 0.2090 | 3.0114695 | 0.945 | -5.6935 | 6.1115 |
|  | 2013 | 0.1568 | 3.0114695 | 0.958 | -5.7457 | 6.0593 |
|  | 2015 | 0.1704 | 3.0114695 | 0.955 | -5.7321 | 6.0730 |
|  | 2016 | 0.2060 | 3.0114695 | 0.945 | -5.6965 | 6.1085 |
|  | 2017 | -0.0161 | 3.0114695 | 0.996 | -5.9187 | 5.8864 |
|  | 2018 | -8.2036* | 3.0114695 | 0.006 | -14.1062 | -2.3011 |
| 2015 | 2012 | 0.0385 | 3.0114695 | 0.990 | -5.8639 | 5.9411 |
|  | 2013 | -0.0136 | 3.0114695 | 0.996 | -5.9161 | 5.8889 |
|  | 2014 | -0.1704 | 3.0114695 | 0.955 | -6.0730 | 5.7321 |
|  | 2016 | 0.0355 | 3.0114695 | 0.991 | -5.8669 | 5.9381 |
|  | 2017 | -0.1865 | 3.0114695 | 0.951 | -6.0891 | 5.7159 |
|  | 2018 | -8.3741* | 3.0114695 | 0.005 | -14.2766 | -2.4715 |
| 2016 | 2012 | 0.0030 | 3.0114695 | 0.999 | -5.8995 | 5.9055 |
|  | 2013 | -0.0492 | 3.0114695 | 0.987 | -5.9517 | 5.8533 |
|  | 2014 | -0.2060 | 3.0114695 | 0.945 | -6.1085 | 5.6965 |
|  | 2015 | -0.0355 | 3.0114695 | 0.991 | -5.9381 | 5.8669 |
|  | 2017 | -0.2221 | 3.0114695 | 0.941 | -6.1247 | 5.6803 |
|  | 2018 | -8.4097* | 3.0114695 | 0.005 | -14.3122 | -2.5071 |
| 2017 | 2012 | 0.2251 | 3.0114695 | 0.940 | -5.6773 | 6.1277 |
|  | 2013 | 0.1729 | 3.0114695 | 0.954 | -5.7296 | 6.0755 |
|  | 2014 | 0.0161 | 3.0114695 | 0.996 | -5.8864 | 5.9187 |
|  | 2015 | 0.1865 | 3.0114695 | 0.951 | -5.7159 | 6.0891 |
|  | 2016 | 0.2221 | 3.0114695 | 0.941 | -5.6803 | 6.1247 |
|  | 2018 | -8.1875* | 3.0114695 | 0.007 | -14.0900 | -2.2849 |
| 2018 | 2012 | 8.4127* | 3.0114695 | 0.005 | 2.5101 | 14.3152 |
|  | 2013 | 8.3604* | 3.0114695 | 0.006 | 2.4579 | 14.2630 |
|  | 2014 | 8.2036* | 3.0114695 | 0.006 | 2.3011 | 14.1062 |
|  | 2015 | 8.3741* | 3.0114695 | 0.005 | 2.4715 | 14.2766 |
|  | 2016 | 8.4097* | 3.0114695 | 0.005 | 2.5071 | 14.3122 |
|  | 2017 | 8.1875* | 3.0114695 | 0.007 | 2.2849 | 14.0900 |

*. The mean difference is significant at the 0.05 level.

**Appendix 4.** Post Hoc Tests (LSD) for multiple Comparisons: ROA

| **(I) Time** | **(J) Time** | **Mean Difference (I-J)** | **Std. Error** | **Sig.** | **95% Confidence Interval** | |
| --- | --- | --- | --- | --- | --- | --- |
|  |  |  |  |  | **Lower Bound** | **Upper Bound** |
| 2012 | 2013 | -0.0077 | 0.0104040 | .457 | -0.0281 | 0.0126 |
|  | 2014 | 0.0058 | 0.0104040 | .571 | -0.0145 | 0.0262 |
|  | 2015 | -0.0331* | 0.0104040 | .001 | -0.0535 | -0.0127 |
|  | 2016 | -0.0196 | 0.0104040 | .059 | -0.0400 | 0.0007 |
|  | 2017 | -0.0533* | 0.0104040 | .000 | -0.0737 | -0.0329 |
|  | 2018 | -0.0453* | 0.0104040 | .000 | -0.0657 | -0.0250 |
| 2013 | 2012 | 0.0077 | 0.0104040 | .457 | -0.0126 | 0.0281 |
|  | 2014 | 0.0136 | 0.0104040 | .190 | -0.0067 | 0.0340 |
|  | 2015 | -0.0253* | 0.0104040 | .015 | -0.0457 | -0.0049 |
|  | 2016 | -0.0119 | 0.0104040 | .252 | -0.0323 | 0.0084 |
|  | 2017 | -0.0456* | 0.0104040 | .000 | -0.0660 | -0.0252 |
|  | 2018 | -0.0376* | 0.0104040 | .000 | -0.0580 | -0.0172 |
| 2014 | 2012 | -0.0058 | 0.0104040 | .571 | -0.0262 | 0.0145 |
|  | 2013 | -0.0136 | 0.0104040 | .190 | -0.0340 | 0.0067 |
|  | 2015 | -0.0390* | 0.0104040 | .000 | -0.0593 | -0.0186 |
|  | 2016 | -0.0255* | 0.0104040 | .014 | -0.0459 | -0.0051 |
|  | 2017 | -0.0592* | 0.0104040 | .000 | -0.0796 | -0.0388 |
|  | 2018 | -0.0512* | 0.0104040 | .000 | -0.0716 | -0.0308 |
| 2015 | 2012 | 0.0331* | 0.0104040 | .001 | 0.0127 | 0.0535 |
|  | 2013 | 0.0253* | 0.0104040 | .015 | 0.0049 | 0.0457 |
|  | 2014 | 0.0390* | 0.0104040 | .000 | 0.0186 | 0.0593 |
|  | 2016 | 0.0134 | 0.0104040 | .196 | -0.0069 | 0.0338 |
|  | 2017 | -0.0202 | 0.0104040 | .052 | -0.0406 | 0.0001 |
|  | 2018 | -0.0122 | 0.0104040 | .238 | -0.0326 | 0.0081 |
| 2016 | 2012 | 0.0196 | 0.0104040 | .059 | -0.0007 | 0.0400 |
|  | 2013 | 0.0119 | 0.0104040 | .252 | -0.0084 | 0.0323 |
|  | 2014 | 0.0255* | 0.0104040 | .014 | 0.0051 | 0.0459 |
|  | 2015 | -0.0134 | 0.0104040 | .196 | -0.0338 | 0.0069 |
|  | 2017 | -0.0337* | 0.0104040 | .001 | -0.0541 | -0.0133 |
|  | 2018 | -0.0257* | 0.0104040 | .013 | -0.0461 | -0.0053 |
| 2017 | 2012 | 0.0533* | 0.0104040 | .000 | 0.0329 | 0.0737 |
|  | 2013 | 0.0456* | 0.0104040 | .000 | 0.0252 | 0.0660 |
|  | 2014 | 0.0592* | 0.0104040 | .000 | 0.0388 | 0.0796 |
|  | 2015 | 0.0202 | 0.0104040 | .052 | -0.0001 | 0.0406 |
|  | 2016 | 0.0337* | 0.0104040 | .001 | 0.0133 | 0.0541 |
|  | 2018 | 0.0079 | 0.0104040 | .444 | -0.0124 | 0.0283 |
| 2018 | 2012 | 0.0453* | 0.0104040 | .000 | 0.0250 | 0.0657 |
|  | 2013 | 0.0376* | 0.0104040 | .000 | 0.0172 | 0.0580 |
|  | 2014 | 0.0512* | 0.0104040 | .000 | 0.0308 | 0.0716 |
|  | 2015 | 0.0122 | 0.0104040 | .238 | -0.0081 | 0.0326 |
|  | 2016 | 0.0257* | 0.0104040 | .013 | 0.0053 | 0.0461 |
|  | 2017 | -0.0079 | 0.0104040 | .444 | -0.0283 | 0.0124 |

*. The mean difference is significant at the 0.05 level.

**Appendix 5.** Post Hoc Tests (LSD) for multiple Comparisons: FL

| **(I) Time** | **(J) Time** | **Mean Difference (I-J)** | **Std. Error** | **Sig.** | **95% Confidence Interval** | |
| --- | --- | --- | --- | --- | --- | --- |
|  |  |  |  |  | **Lower Bound** | **Upper Bound** |
| 2012 | 2013 | -0.4222 | 0.3208444 | 0.188 | -1.0510 | 0.2066 |
|  | 2014 | 0.0569 | 0.3208444 | 0.859 | -0.5719 | 0.6857 |
|  | 2015 | 0.7154* | 0.3208444 | 0.026 | 0.0865 | 1.3442 |
|  | 2016 | 0.5538 | 0.3208444 | 0.084 | -0.0750 | 1.1827 |
|  | 2017 | -2.0407* | 0.3208444 | 0.000 | -2.6696 | -1.4119 |
|  | 2018 | -0.7913080* | 0.3208444 | 0.014 | -1.4201 | -0.1624 |
| 2013 | 2012 | 0.4222 | 0.3208444 | 0.188 | -0.2066 | 1.0510 |
|  | 2014 | 0.4791 | 0.3208444 | 0.135 | -0.1497 | 1.1079 |
|  | 2015 | 1.1376* | 0.3208444 | 0.000 | 0.5087 | 1.7664 |
|  | 2016 | 0.9760* | 0.3208444 | 0.002 | 0.3472 | 1.6049 |
|  | 2017 | -1.6185* | 0.3208444 | 0.000 | -2.2474 | -0.9897 |
|  | 2018 | -0.3690 | 0.3208444 | 0.250 | -0.9979 | 0.2597 |
| 2014 | 2012 | -0.0569 | 0.3208444 | 0.859 | -0.6857 | 0.5719 |
|  | 2013 | -0.4791 | 0.3208444 | 0.135 | -1.1079 | 0.1497 |
|  | 2015 | 0.6584* | 0.3208444 | 0.040 | 0.0296 | 1.2873 |
|  | 2016 | 0.4969 | 0.3208444 | 0.121 | -0.1319 | 1.1258 |
|  | 2017 | -2.0977* | 0.3208444 | 0.000 | -2.7265 | -1.4688 |
|  | 2018 | -0.8482* | 0.3208444 | 0.008 | -1.4770 | -0.2193 |
| 2015 | 2012 | -0.7154* | 0.3208444 | 0.026 | -1.3442 | -0.0865 |
|  | 2013 | -1.1376* | 0.3208444 | 0.000 | -1.7664 | -0.5087 |
|  | 2014 | -0.6584* | 0.3208444 | 0.040 | -1.2873 | -0.02962 |
|  | 2016 | -0.1615 | 0.3208444 | 0.615 | -0.7904 | 0.4673 |
|  | 2017 | -2.7562* | 0.3208444 | 0.000 | -3.3850 | -2.1273 |
|  | 2018 | -1.5067* | 0.3208444 | 0.000 | -2.1355 | -0.8778 |
| 2016 | 2012 | -0.5538 | 0.3208444 | 0.084 | -1.1827 | 0.0750 |
|  | 2013 | -0.9760* | 0.3208444 | 0.002 | -1.6049 | -0.3472 |
|  | 2014 | -0.4969 | 0.3208444 | 0.121 | -1.1258 | 0.1319 |
|  | 2015 | 0.1615 | 0.3208444 | 0.615 | -0.4673 | 0.7904 |
|  | 2017 | -2.5946* | 0.3208444 | 0.000 | -3.2235 | -1.9657 |
|  | 2018 | -1.3451* | 0.3208444 | 0.000 | -1.9740 | -0.7163 |
| 2017 | 2012 | 2.0407* | 0.3208444 | 0.000 | 1.4119 | 2.6696 |
|  | 2013 | 1.6185* | 0.3208444 | 0.000 | 0.9897 | 2.2474 |
|  | 2014 | 2.0977* | 0.3208444 | 0.000 | 1.4688 | 2.7265 |
|  | 2015 | 2.7562* | 0.3208444 | 0.000 | 2.1273 | 3.3850 |
|  | 2016 | 2.5946* | 0.3208444 | 0.000 | 1.9657 | 3.2235 |
|  | 2018 | 1.2494* | 0.3208444 | 0.000 | 0.6206 | 1.8783 |
| 2018 | 2012 | 0.7913* | 0.3208444 | 0.014 | 0.1624 | 1.4201 |
|  | 2013 | 0.3690 | 0.3208444 | 0.250 | -0.2597 | 0.9979 |
|  | 2014 | 0.8482* | 0.3208444 | 0.008 | 0.2193 | 1.4770 |
|  | 2015 | 1.5067* | 0.3208444 | 0.000 | 0.8778 | 2.1355 |
|  | 2016 | 1.3451* | 0.3208444 | 0.000 | 0.7163 | 1.9740 |
|  | 2017 | -1.2494* | 0.3208444 | 0.000 | -1.8783 | -0.6206 |

*. The mean difference is significant at the 0.05 level.

**Appendix 6.** Post Hoc Tests (LSD) for multiple Comparisons: PBR

| **(I) Time** | **(J) Time** | **Mean Difference (I-J)** | **Std. Error** | **Sig.** | **95% Confidence Interval** | |
| --- | --- | --- | --- | --- | --- | --- |
|  |  |  |  |  | **Lower Bound** | **Upper Bound** |
| 2012 | 2013 | -0.8671 | 0.4975802 | 0.081 | -1.8424 | 0.1081 |
|  | 2014 | -0.1340 | 0.4975802 | 0.788 | -1.1093 | 0.8411 |
|  | 2015 | -0.6911 | 0.4975802 | 0.165 | -1.6663 | 0.2841 |
|  | 2016 | 0.5740 | 0.4975802 | 0.249 | -0.4011 | 1.5493 |
|  | 2017 | -2.6200* | 0.4975802 | 0.000 | -3.5953 | -1.6448 |
|  | 2018 | -1.5640* | 0.4975802 | 0.002 | -2.5392 | -0.5887 |
| 2013 | 2012 | 0.8671 | 0.4975802 | 0.081 | -0.1081 | 1.8424 |
|  | 2014 | 0.7330 | 0.4975802 | 0.141 | -0.2422 | 1.7083 |
|  | 2015 | 0.1760 | 0.4975802 | 0.723 | -0.7992 | 1.1513 |
|  | 2016 | 1.4412* | 0.4975802 | 0.004 | 0.4659 | 2.4165 |
|  | 2017 | -1.7529* | 0.4975802 | 0.000 | -2.7281 | -0.7776 |
|  | 2018 | -0.6968 | 0.4975802 | 0.161 | -1.6721 | 0.2784 |
| 2014 | 2012 | 0.1340 | 0.4975802 | 0.788 | -0.8411 | 1.1093 |
|  | 2013 | -0.7330 | 0.4975802 | 0.141 | -1.7083 | 0.2422 |
|  | 2015 | -0.5570 | 0.4975802 | 0.263 | -1.5322 | 0.4182 |
|  | 2016 | 0.7081 | 0.4975802 | 0.155 | -0.2670 | 1.6834 |
|  | 2017 | -2.4859* | 0.4975802 | 0.000 | -3.4612 | -1.5107 |
|  | 2018 | -1.4299* | 0.4975802 | 0.004 | -2.4051 | -0.4546 |
| 2015 | 2012 | 0.6911 | 0.4975802 | 0.165 | -0.2841 | 1.6663 |
|  | 2013 | -0.1760 | 0.4975802 | 0.723 | -1.1513 | 0.7992 |
|  | 2014 | 0.5570 | 0.4975802 | 0.263 | -0.4182 | 1.5322 |
|  | 2016 | 1.2651* | 0.4975802 | 0.011 | 0.2899 | 2.2404 |
|  | 2017 | -1.9289* | 0.4975802 | 0.000 | -2.9042 | -0.9536 |
|  | 2018 | -0.8729 | 0.4975802 | 0.079 | -1.8481 | 0.1023 |
| 2016 | 2012 | -0.5740 | 0.4975802 | 0.249 | -1.5493 | 0.4011 |
|  | 2013 | -1.4412* | 0.4975802 | 0.004 | -2.4165 | -0.4659 |
|  | 2014 | -0.7081 | 0.4975802 | 0.155 | -1.6834 | 0.2670 |
|  | 2015 | -1.2651* | 0.4975802 | 0.011 | -2.2404 | -0.2899 |
|  | 2017 | -3.1941* | 0.4975802 | 0.000 | -4.1694 | -2.2188 |
|  | 2018 | -2.1381* | 0.4975802 | 0.000 | -3.1133 | -1.1628 |
| 2017 | 2012 | 2.6200* | 0.4975802 | 0.000 | 1.6448 | 3.5953 |
|  | 2013 | 1.7529* | 0.4975802 | 0.000 | 0.7776 | 2.7281 |
|  | 2014 | 2.4859* | 0.4975802 | 0.000 | 1.5107 | 3.4612 |
|  | 2015 | 1.9289* | 0.4975802 | 0.000 | 0.9536 | 2.9042 |
|  | 2016 | 3.1941* | 0.4975802 | 0.000 | 2.2188 | 4.1694 |
|  | 2018 | 1.0560* | 0.4975802 | 0.034 | 0.0807 | 2.0313 |
| 2018 | 2012 | 1.5640* | 0.4975802 | 0.002 | 0.5887 | 2.5392 |
|  | 2013 | 0.6968 | 0.4975802 | 0.161 | -0.2784 | 1.6721 |
|  | 2014 | 1.4299* | 0.4975802 | 0.004 | 0.4546 | 2.4051 |
|  | 2015 | 0.8729 | 0.4975802 | 0.079 | -0.1023 | 1.8481 |
|  | 2016 | 2.1381* | 0.4975802 | 0.000 | 1.1628 | 3.1133 |
|  | 2017 | -1.0560* | 0.4975802 | 0.034 | -2.0313 | -0.0807 |

*. The mean difference is significant at the 0.05 level.

**Appendix 7.** Pearson Correlations

|  | | **ROE** | **ROA** | **FL** | **PBR** |
| --- | --- | --- | --- | --- | --- |
| ROE | Pearson Correlation | 1 | -0.005 | 0.017** | 0.543** |
|  | Sig. (2-tailed) |  | 0.326 | 0.001 | 0.000 |
|  | N | 37709 | 37709 | 37709 | 37709 |
| ROA | Pearson Correlation | -0.005 | 1 | 0.031** | 0.017** |
|  | Sig. (2-tailed) | 0.326 |  | 0.000 | 0.001 |
|  | N | 37709 | 37709 | 37709 | 37709 |
| FL | Pearson Correlation | 0.017** | 0.031** | 1 | 0.298** |
|  | Sig. (2-tailed) | 0.001 | 0.000 |  | 0.000 |
|  | N | 37709 | 37709 | 37709 | 37709 |
| PBR | Pearson Correlation | 0.543** | 0.017** | 0.298** | 1 |
|  | Sig. (2-tailed) | 0.000 | 0.001 | 0.000 |  |
|  | N | 37709 | 37709 | 37709 | 37709 |

**. Correlation is significant at the 0.01 level (2-tailed).
